# Beneficial Effect of *Ocimum sanctum* (Linn) against Monocrotaline-Induced Pulmonary Hypertension in Rats

**DOI:** 10.3390/medicines5020034

**Published:** 2018-04-17

**Authors:** Himanshu Meghwani, Pankaj Prabhakar, Soheb A. Mohammed, Pamila Dua, Sandeep Seth, Milind P. Hote, Sanjay K. Banerjee, Sudheer Arava, Ruma Ray, Subir Kumar Maulik

**Affiliations:** 1 Department of Pharmacology, All India Institute of Medical Sciences (AIIMS), New Delhi 110029, India; meghwani.himanshu@gmail.com (H.M.); pprabhakaraiims@gmail.com (P.P.); drpamiladua@gmail.com (P.D.); 2 Drug Discovery Research Center (DDRC), Translational Health Science and Technology Institute (THSTI), Faridabad 121001, India; sohebmsa@gmail.com (S.A.M.); skbanerjee@thsti.res.in (S.K.B.); 3 Department of Cardiology, AIIMS, New Delhi 11029, India; drsandeepseth@hotmail.com; 4 Department of CTVS, AIIMS, New Delhi 110029, India; mphaiims@hotmail.com; 5 Department of Pathology, AIIMS, New Delhi 110029, India; aravaaiims@gmail.com (S.A.); rumasrc2@hotmail.co (R.R.)

**Keywords:** *Ocimum sanctum*, pulmonary hypertension, echocardiography, percentage medial wall thickness, Nox

## Abstract

**Background:** The study was designed to explore any beneficial effect of Ocimum sanctum (Linn) (OS) in experimental pulmonary hypertension (PH) in rats. OS is commonly known as “holy basil” and “Tulsi” and is used in the Indian System of Medicine as antidiabetic, antioxidant, hepatoprotective, adaptogenic, and cardioprotective. **Methods:** Monocrotaline (MCT) administration caused development of PH in rats after 28 days and rats were observed for 42 days. Treatments (sildenafil; 175 µg/kg, OS; 200 mg/kg) were started from day 29 after the development of PH and continued for 14 days. Parameters to assess the disease development and effectiveness of interventions were echocardiography, right and left ventricular systolic pressures, and right ventricular end diastolic pressure, percentage medial wall thickness (%MWT) of pulmonary artery, oxidative stress markers in lung tissue, NADPH oxidase (Nox-1) protein expression in lung, and mRNA expression of Bcl2 and Bax in right ventricular tissue. **Results:** OS (200 mg/kg) treatment ameliorated increased lung weight to body weight ratio, right ventricular hypertrophy, increased RVSP, and RVoTD/AoD ratio. Moreover, OS treatment decreases Nox-1 expression and increases expression of Bcl2/Bax ratio caused by MCT. **Conclusion:** The present study demonstrates that OS has therapeutic ability against MCT-induced PH in rat which are attributed to its antioxidant effect. The effect of OS was comparable with sildenafil.

## 1. Introduction

*Ocimum sanctum* (OS) (Linn) (an auspicious plant called “holy basil” or “*Tulsi*”) is one of the medicinal plants widely used in the Indian System of Medicine. Various animal and clinical studies have validated its antioxidant [1], immuno-modulatory [2], anti-inflammatory [3], analgesic [4], antimicrobial [5], anti-ischemic [6], antihypertensive [7], and cardioprotective [8] properties. Constituents of the hydroalcoholic extract of leaves of OS include flavonoids (orientin and vicenin) which are known to increase glutathione (GSH) and other antioxidant enzymes and scavenge lipid peroxides [9]. The leaves of OS have been shown to possess immunomodulatory [10], antihyperlipidemic, and antioxidant [11] activity in experimental animals. We have also reported that OS caused significant protection against isoproterenol-induced myocardial necrosis through a unique property of enhancement of endogenous antioxidants [8] and protected rat heart from chronic restraint stress-induced cardiac changes [12]. The antiproliferative effect of OS in carcinogenesis was mediated by modulating key proteins involved in cellular proliferation, apoptosis, angiogenesis, and invasion [13]. Thus, the reported cardio-protective, antioxidant, and anti-proliferative properties of the hydroalcoholic extract of leaves of OS led to our hypothesis that it might have a salutary effect in pulmonary hypertension.

Pulmonary hypertension (PH) is defined and diagnosed hemodynamically as mean pulmonary artery pressure (mPAP) of more than 25 mm of Hg at rest along with normal pulmonary capillary wedge pressure and pulmonary vascular resistance of more than 3 woods unit after right heart catheterization [14]. Even after 20 years of extensive research, the mortality associated with this disease is still high. Survival in a cohort study from Denmark was approximately 86% after 1 year, 73% after 3 years, and 65% after 5 years in spite of utilizing all available treatment [15]. The pathology involves apoptosis-resistant proliferation of pulmonary artery endothelial and smooth cells (PAEC and PASMC) which leads to narrowing of the arteriolar lumen and compensatory right ventricular hypertrophy and subsequent right ventricular failure which is the main cause of death.

The molecular mechanisms of initiation of lumen narrowing are not yet well identified, but progression involves imbalance between vasodilators (nitric oxide and prostacyclins) and vasoconstrictors (endothelin-1 and serotonin), apoptosis-resistant proliferation of pulmonary artery smooth muscle cell and endothelial cells (PASMC and PAEC), inflammatory cytokines and chemokines, and oxidative stress (reactive oxygen and nitrogen species) [16].

It has been demonstrated that reactive oxygen species (ROS) produce detrimental effects in PH [17]. Nicotinamide adenine dinucleotide phosphate (NADPH) oxidase (Nox) is an enzyme responsible for ROS and RNS production. Nox-1 isoform was found to be increased in pulmonary arteries of rats after monocrotaline administration [18], furthermore, this amplified ROS production is implicated in proliferation of PASMC in humans [19] as well as in the experimental model [20].

Therefore, the present study was designed to evaluate the therapeutic potential of a hydroalcoholic extract of leaves of OS in monocrotaline-induced PH in rats.

## 2. Materials and Methods

### 2.1. Ethical Aspects

The study protocol was approved by the Institutional Animal Ethics Committee (IAEC), All India Institute of Medical Sciences (AIIMS), New Delhi, India (564/IAEC/10). All experimental protocols were performed in compliance with the National Institutes of Health (NIH) guidelines for the care and use of laboratory animals (NIH Publication no. 85723, revised 1996). Ethical approval code: 564/IAEC/10, Date of approval: 24 September 2012.

### 2.2. Experimental Animals

Male Wistar rats (150–200 g, 10–12 weeks) were used for the study and were maintained under standard laboratory conditions (temperature 25 ± 2 °C; relative humidity 50 ± 15%; and natural dark/light cycle). A commercial standard rat chow diet (Ashirwad, Chandigarh, India) and water were provided *ad libitum*.

### 2.3. Drugs and Chemicals

A hydroalcoholic extract of leaves of OS (Dabur India Limited, Ghaziabad, India) was used in a dose of 200 mg/kg/day and dose was selected from an earlier study from our laboratory [8]. Sildenafil citrate salt (Sigma-Aldrich, ‎St. Louis, MO,‎ USA) was used in this study as a standard comparator drug. A dose of 175 µg/kg/day [21] of sildenafil dissolved in distilled water was administered orally.

Monocrotaline (MCT; Sigma-Aldrich, USA) was weighed and dissolved in 1.0 N HCl which was then neutralized to pH 7.4 by adding 1.0 N NaOH and distilled water was added to make up the volume. A single injection of 50 mg/kg of monocrotaline known to stimulate PH was administered subcutaneously [22,23].

All other chemicals used for experiments were of analytical grade and purchased from Sigma-Aldrich Co., USA.

### 2.4. Experimental Design

Rats were randomly assigned to different experimental groups (*n* = 6):

1. Control: Normal saline was administered subcutaneously once in a dose of 1.0 mL/kg and observed for 42 days.
2. MCT: Monocrotaline was administered subcutaneously in a single dose of 50 mg/kg on day 1 and observed for 42 days.
3. MCT + SIL: Rats were injected MCT (50 mg/kg; sc) followed by sildenafil 175 µg/kg/day orally once daily started from day 29 after MCT administration and continued for 14 days.
4. MCT + OS 200: Rats were injected MCT (50 mg/kg; sc) followed by OS (200 mg/kg/day) (the more effective dose taken from previous preventive study) orally started from day 29 after MCT administration and continued for 14 days.

Monocrotaline was administered to rats after baseline echocardiography on day 1 and were observed for 28 days. Echocardiography was repeated to confirm development of PH before and after OS or sildenafil administration (day 29 to day 42), while control and MCT groups were observed till day 42. At the end of the study protocol and before surgical procedure, echocardiography was done to evaluate the changes among the groups.

### 2.5. Parameters Done

#### 2.5.1. Echocardiography

Rats were anaesthetized with 50 mg/kg intraperitoneal injection of sodium pentobarbitone reconstituted in normal saline [23], body temperature was maintained at 37 °C with warmer. Chest was shaved using hair remover cream. Transthoracic closed-chest echocardiography was done using a mechanical transducer centered on 12 MHz probe (Philips, Andover, MA, USA). M-mode and 2-dimensional images were captured by aligning the probe in parasternal long and short axis views, which were later used to measure right ventricle outflow tract dimension (RVoTD), aortic outflow dimension (AoD), their ratio (RVoTD/AoD) [24], and left ventricular ejection fraction (%LVEF) [25]. Doppler tracings were recorded at a sweep speed of 150 mm/s. The following variables were measured: pulmonary artery acceleration time (PAAT, defined as the time from the onset of flow to peak velocity by pulsed-wave doppler recording), right ventricular ejection time (ET, the time from the onset to the termination of pulmonary flow) [26] and heart rate was also measured [27] PAAT/ET is inversely and linearly proportional to right ventricular systolic pressure (RVSP) in rats as described earlier [28]. Images were captured for offline measurements and were analyzed by another author (S.S.) who was blinded to the groups.

#### 2.5.2. Hemodynamic Parameters

Under sodium pentobarbitone anesthesia [29], rats were prepared for measurement of hemodynamic parameters. Approximately 1.0 cm midline incision was made in the neck. Rats were intubated with a 14G polythene cannula using guidewire. The cannula was connected to a small animal ventilator (Columbus instruments, Columbus, OH, USA) which was set to deliver 60 breaths per minute and tidal volume of 8.0 mL/kg [30]. A median sternotomy was performed to expose the heart and position was maintained by using adjustable chest retractors. A 24G pediatric cannula that attached to an electronic pressure transducer was introduced into the right and left ventricles. Right ventricular systolic pressure (RVSP), a marker of systolic pulmonary artery pressure [31] and right ventricular end diastolic pressure (RVeDP), a measure of diastolic function of right ventricle, were measured [32]. Another 24G cannula was inserted into the left ventricular cavity through the posterior part of the apex to measure the left ventricular systolic pressure (LVSP) [33] using a digital data acquisition system (Biopac, Goleta, CA, USA). The pressure tracings were digitally recorded and stored for offline analysis on desktop using Acqknoweledge 4.3 software (Biopac, Goleta, CA, USA). At the end of hemodynamic measurements, rats were sacrificed by an overdose of sodium pentobarbitone (120 mg/kg). Heart and lung tissue were separated and weighed.

#### 2.5.3. Morphometric Parameters

Body weight was measured on day 1, day 28 and day 42 with the help of electronic balance (Oras Tech, Haryana, India).

#### 2.5.4. Right Ventricular Hypertrophy Index (RVH Index)

Right ventricular (RV) free wall weight was separated from left ventricle and inter-ventricular septum (LV + IVS) and the two were weighed separately. The ratio of right ventricular weight to heart weight (RV/HW) and ratio of right ventricular weight to left ventricular plus inter-ventricular septum weight [(RV/(LV + IVS) also called as Fulton’s Index] were calculated [34]. Thereafter, the heart tissue was snap frozen in liquid nitrogen and stored at −80 °C for RT-PCR studies.

#### 2.5.5. Lung Tissue

Both lobes (right and left) of the lung were separated and weighed excluding the bronchi and trachea, and the ratio of total lung weight to body weight was estimated (TLW/BW). Right middle lobe was preserved in 10% formalin for histopathology, while other lobes were snap frozen in liquid nitrogen and stored at −80 °C for oxidative stress markers and western blotting. Wet weight of left lobe of lung was measured then it was dried at 70 °C in oven for 3 days until the weight becomes constant and the percentage of dry weight to wet weight was calculated [35].

#### 2.5.6. Histopathology

After 48–72 h in formalin, the lung tissue samples get fixed and then processed under routine automated histokinetic processor. Paraffin blocks were prepared, 4–5 µm thick sections were cut and stained for routine hematoxylin and eosin stain. Special histochemical stain (Elastic Van Gieson) was carried out to highlight the elastic lamina. Thirty vessels were randomly selected from each section [36]. The vessels were grouped according to their external diameter into two groups <100 µm and >100 µm to ascertain the changes in the smaller and larger arterioles [37]. Percentage medial wall thickness was measured using the formula %MWT = medial wall thickness/external diameter × 100 [38], using Olympus imaging software (Cellsens, Tokyo, Japan).

#### 2.5.7. Oxidative Stress Markers in Lung Tissue

Lung tissue was homogenized in 0.1 M phosphate buffer (pH-7.4) and centrifuged at 10,000× *g* at 4 °C for 15 min (REMI, Maharashtra, India). Dilution of 1:10 (*w*/*v*) was used for homogenization. Supernatant was taken for oxidative stress markers.

##### Thiobarbituric Acid Reactive Substances (TBARS) in Lung Homogenate

TBARS was estimated by the method described by Ohkawa et al. [39]. Briefly, the reagents acetic acid 1.5 mL (20%) pH-3.5, 1.5 mL thiobarbituric acid (0.8%), and 0.2 mL sodium lauryl sulphate (8.1%), 0.6 mL distilled water were added to 0.2 mL of sample. The mixture was heated at 95 °C for 60 min. The mixture was cooled at room temperature and 5.0 mL of n-butanol:pyridine (15:1) and 1.0 mL distilled water were added. After centrifugation at 5000 rpm for 10 min at room temperature, the upper pink colored organic layer was separated, and absorbance was measured at 532 nm using a spectrophotometer (Specord 200, Analytik Jena, Germany).

##### Reduced Glutathione (GSH) Levels in Lung Homogenate

Reduced glutathione was estimated by the method described earlier [40]. Briefly, 0.1 mL sample was added to 2.0 mL phosphate buffer (pH-8.4), 0.5 mL 5′5 dithiobis (2-nitrobenzoic acid) (DTNB) and 0.4 mL of double distilled water. The mixture was vortexed and the absorbance was read at 412 nm within 15 min using a spectrophotometer (Specord 200, Analytik Jena, Germany).

##### Catalase Levels in Lung Homogenate

Catalase was estimated as described earlier [41]. Briefly, 0.05 mL sample was added to 1.95 mL of 50 mM phosphate buffer and 1.0 mL H_2_O_2_ was added and extinction was read at 240 nm at 15 s interval for a total of 60 s by spectrophotometer (Specord 200, Analytik Jena, Germany).

##### Superoxide Dismutase (SOD) Enzyme Levels in Lung Homogenate

Superoxide dismutase was estimated by the method described earlier [42]. Briefly, the reagents, sodium pyrophosphate buffer 1.2 mL (0.052 M, pH-8.3), 0.1 mL phenazinemethosulphate (186 µM), 0.3 mL nitro blue tetrazolium (300 µM), and 0.2 mL NADH (780 µM) were added to 0.1 mL of sample. The mixture was incubated for 90 s at 30 °C. Then 4.0 mL of n-butanol and 1.0 mL of acetic acid were added. After centrifugation at 4000 rpm for 10 min at room temperature, the organic layer was separated and absorbance was measured at 560 nm using a spectrophotometer (Specord 200, Analytik Jena, Germany).

#### 2.5.8. Western Blot Analysis of Lung NADPH Oxidase (Nox-1)

Total protein extraction and immunoblotting were performed in RIPA buffer enriched with protease and phosphatase inhibitors. Protein concentration was determined by the Bradford assay. An equal amount (30 µg) of protein was used for the immunoblotting and separated by sodium dodecyl sulfate polyacrylamide gel electrophoresis (SDS-PAGE). After electrophoresis, protein was transferred to PVDF membranes (Millipore, Burlington, MA, USA). The membranes were then blocked in Tris-buffered saline Tween-20 (TBS-T; 10 mM Tris, pH 7.5, 150 mM NaCl, 0.05% Tween-20) and 5% non-fat dry milk for 1 h and subsequently washed and incubated with primary antibodies in TBST at 4 °C for overnight. The titer of polyclonal antibody (anti-Nox-1, Abcam, Cambridge, UK) used was 1:1000. After washing with TBST, the membrane was incubated with goat anti-rabbit IgG-HRP (1:4000 dilution, Cell Signaling Technology, Danvers, MA, USA, # SC 2004) antibody with 2.5% non-fat dry milk at room temperature for 2 h. After washing with TBS-T, immunoreactions were visualized with a chemiluminescence detection kit (Prod No-34080, Super signal^®^ west Pico chemiluminescent substrate, Thermo Scientific, Waltham, MA, USA). Then, the blots were exposed to the (Bio-Rad, Hercules, CA, USA) chemiluminescence detection system and developed. Expression was normalized with loading control. Identification of band intensity was performed using the Image J Software (NIH, Bethesda, MD, USA).

#### 2.5.9. Reverse Transcriptase Polymerase Chain Reaction (RT-PCR)

Total RNA was isolated from RV of rat heart using TRIzol (Sigma). Reverse transcriptase reactions were performed for cDNA synthesis according to the method described earlier [43]. Primers (Table 1) for gene expression analysis were designed using published sequence information, avoiding regions of homology with other genes. PCR was performed in a 0.2 mL tube containing 2 µL cDNA, 1 µL (10 pmol) rat forward primer and reverse primer, 2.0 µL dNTP (1.25 mM each nucleotide), 2.5 µL 10× PCR Buffer, 0.25 µL Taq polymerase, and 11.25 µL dH_2_O. After denaturation at 95 °C for 5 min, the whole mixture underwent PCR for 32 cycles at 95 °C for 60 s, annealing temperature for 60 s, 72 °C for 60 s. The PCR product separated on 2% agarose gel stained with ethidium bromide. The optical density of different gene bands was measured by the Gel Documentation System (Bio Rad). The RPL-32 gene was used as a reference gene. We evaluated and quantified PCR product by agarose gel electrophoresis. Quantification of bands on the agarose gel was performed using Image J Software (NIH, USA). Fold-change analysis was based on normalizing to RPL-32 transcript levels in each sample.

### 2.6. Statistical Analysis

Results are represented as mean ± standard error of mean (SEM). One-way analysis of variance (ANOVA) followed by Bonferroni multiple comparison test (SPSS-20 software, IBM Analytics, Armonk, NY, USA) was done. The level of statistical significance was set at *p* < 0.05.

## 3. Results

### 3.1. Morphometric Parameters and Right Ventricular Hypertrophy Index (RVH Index)

No significant difference in body weight was observed among the groups at the end of the study.

**Figure 1:**
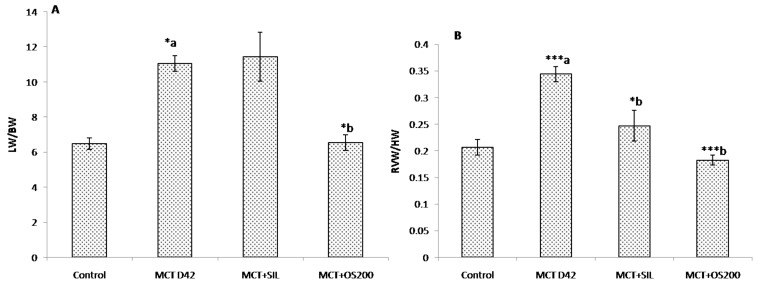

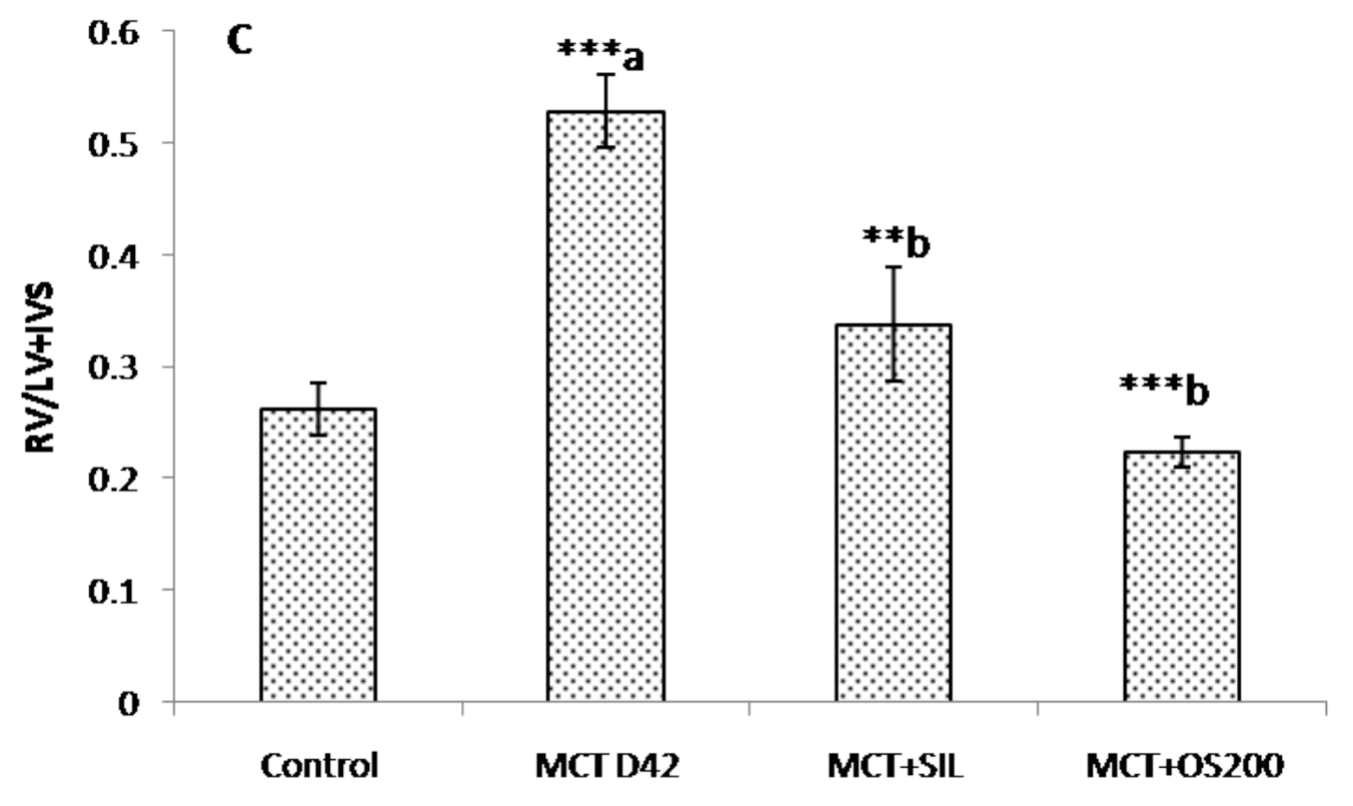
Effect of *O. sanctum* (OS) on (**A**) LW/HW; (**B**) RV/HW; (**C**) Fulton’s index [RV/(LV + IVS)] in MCT-induced PH. Values are expressed as mean ± SEM (*n* = 6). * *p* < 0.05, ** *p* < 0.01, *** *p* < 0.001; a—as compared to control, b—as compared to MCT where; HW: Heart weight, LW: Lung weight, RV: Right ventricular weight, LV: Left ventricular weight, IVS: Interventricular septum weight.

Total lung weight to body weight ratio was found to be significantly increased at 42 days (*p* < 0.05) in the MCT administered group, which was significantly decreased by OS (*p* < 0.05). However, sildenafil did not cause significant change (Figure 1A). Lung % dry/wet weight was found to be same for all the groups.

Similarly, both right ventricular weight to heart weight ratio (*p* < 0.001) and the ratio of right ventricular (RV) weight to left ventricle (LV) plus interventricular septum (IVS) weight (Fulton index) (*p* < 0.001) were increased in MCT administered rats as compared to control (Figure 1B,C). Moreover, sildenafil (*p* < 0.001), and OS (*p* < 0.001) cured (normalized) the parameters of RV hypertrophy (Figure 1).

### 3.2. Hemodynamic Parameters

**Figure 2:**
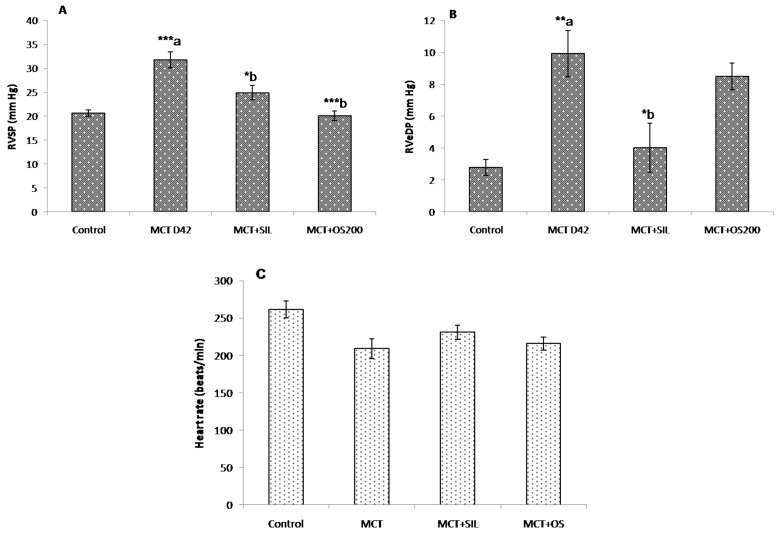
Effect of OS on (**A**) right ventricular systolic pressure; (**B**) right ventricular end diastolic pressure, and (**C**) heart rate in MCT-induced PH. Values are expressed as mean ± SEM (*n* = 6). * *p* < 0.05, ** *p* < 0.01, *** *p* < 0.001; a—as compared to control, b—as compared to MCT.

MCT caused significant increase in right ventricular systolic (RVSP) and end diastolic pressures (RVeDP) at 42 days (Figure 2A,B). OS and sildenafil significantly reduced RVSP in MCT administered rats which were comparable to control while, only sildenafil reduced RVeDP caused by MCT (Figure 2B). There was no significant change observed in heart rate among the groups (Figure 2C). No significant change was observed in left ventricular pressure among all the groups, suggesting no changes in left ventricular functions.

### 3.3. Echocardiography

**Figure 3:**
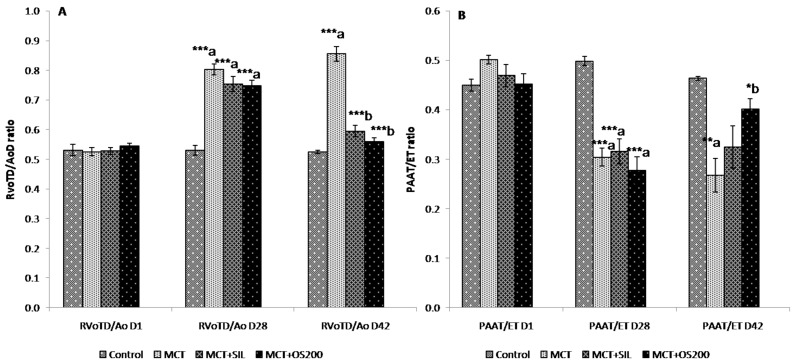
Effect of OS on (**A**) RVoTD/AoD; and (**B**) PAAT/ET ratio in MCT-induced PH. Values are expressed as mean ± SEM (*n* = 6). * *p* < 0.05, ** *p* < 0.01, *** *p* < 0.001; a—as compared to control, b—as compared to MCT.

There was significant increase in RVoTD/AoD ratio [at 28 days (0.82 ± 0.03 vs. 0.56 ± 0.01; *p* < 0.001); and at 42 days (0.85 ± 0.06 vs. 0.53 ± 0.04; *p* < 0.001)] and decrease in PAAT/ET ratio at 28 days [0.32 ± 0.02 vs. 0.50 ± 0.02; *p* < 0.001) and at 42 days (0.27 ± 0.08 vs. 0.49 ± 0.01; *p* < 0.01)] along with systolic notch in pulsed wave doppler images which were typical of pulmonary artery hypertension in MCT administered rats. After 14 days of OS treatment, it ameliorated both the increase in RVoTD/AoD ratio (0.56 ± 0.03 vs. 0.85 ± 0.06; *p* < 0.001) and decrease in PAAT/ET ratio (0.40 ± 0.02 vs. 0.27 ± 0.08; *p* < 0.05) caused by MCT (Figure 3A,B). However, sildenafil cured only the RVoTD/AoD ratio (0.59 ± 0.02 vs. 0.85 ± 0.06; *p* < 0.001) caused by MCT (Figure 3A). There was no significant change in percentage left ventricular ejection fraction (%LVEF) among the groups. In addition, no significant change was observed in heart rate among the groups.

### 3.4. Histopathology

**Figure 4:**
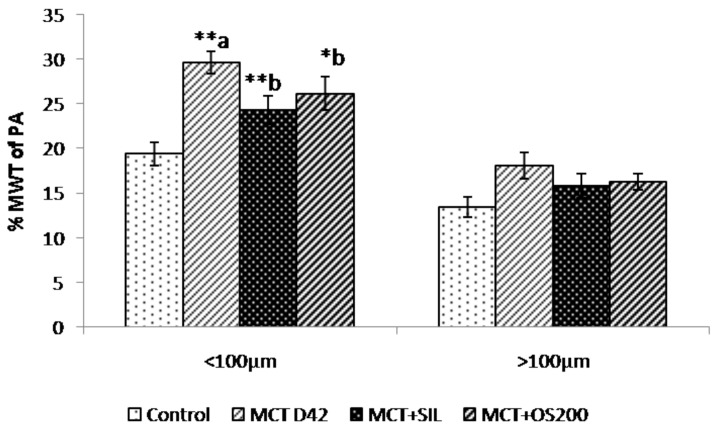
Effect of OS on percentage medial wall thickness of pulmonary artery. Values are expressed as mean ± SEM (*n* = 6). * *p* < 0.05, ** *p* < 0.01; a—as compared to control, b—as compared to MCT.

The percentage medial wall thickness (%MWT) of the pulmonary artery with external diameter less than 100µm was found to be increased in MCT administered rats at 42 days (29.66 ± 1.2% vs. 19.4 ± 1.3%; *p* < 0.01), while the vessels with more than 100 µm external diameter did not show any significant change in %MWT as compared to control. Sildenafil (24.3 ± 1.5% vs. 29.66 ± 1.2%; *p* < 0.01 and OS (26.1 ± 1.8% vs 29.66 ± 1.2%; *p* < 0.05) treatment for 14 days after 28 days administration of MCT significantly decreased the increase in %MWT of pulmonary artery with external diameter less than 100 µm (Figure 4).

### 3.5. Study of Oxidative Stress Parameters in Lung Tissue

#### 3.5.1. Thiobarbituric Acid Reactive Substances (TBARS)

MCT administration caused a significant increase in lung TBARS levels as compared to control at 42 days (155.46 ± 13.4 nmol/mg protein vs. 19.6 ± 2.5 nmol/mg protein; *p* < 0.001). Sildenafil (72.36 ± 12.7 nmol/mg protein; *p* < 0.01) and OS (99.05 ± 20.5 nmol/mg protein vs. 155.46 ± 13.4 nmol/mg protein; *p* < 0.05) treatment significantly reduced lung TBARS levels caused by MCT (Table 2).

#### 3.5.2. Reduced Glutathione (GSH)

There was a significant decrease in lung reduced GSH levels in MCT group as compared to control at 42 days (13.98 ± 2.0 mg/mg protein vs. 27.36 ± 3.0 mg/mg protein; *p* < 0.001). OS and sildenafil administration did not cause significant difference in lung reduced GSH level as compared to MCT administered rats.

#### 3.5.3. Catalase

MCT administration significantly decreased the lung catalase level as compared to control at 42 days (0.21 ± 0.01 U/mg protein vs. 0.85 ± 0.04 U/mg protein; *p* < 0.001). OS treatment after 28 days of MCT administration significantly increased the decrease lung catalase level (Table 2).

#### 3.5.4. Superoxide Dismutase (SOD)

A significant decrease in lung SOD was observed in MCT group at 42 days (35.40 ± 5.7 U/mg protein vs 110.51 ± 10.8 U/mg protein) as compared with control rats. OS and sildenafil treatment did not alleviate the decrease in lung SOD levels caused by MCT (Table 2).

### 3.6. Western Blot Analysis of Lung NADPH Oxidase (Nox-1)

**Figure 5:**
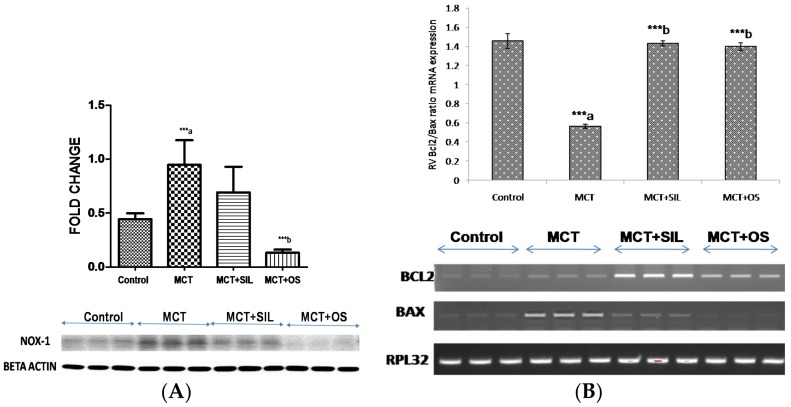
Effect of OS on (**A**) lung Nox-1 protein expression in MCT-induced PH; (**B**) heart mRNA expression of Bcl2/Bax ratio. *** *p* < 0.001; a—as compared to control, b—as compared to MCT. *** *p* < 0.001; a—as compared to control, b—as compared to MCT.

A significant increase in lung Nox-1 protein expression was observed in MCT administered rats as compared to control (*p* < 0.001). OS treatment significantly decreased the protein expression of lung Nox-1 caused by MCT administration (*p* < 0.001). However, sildenafil did not significantly decrease protein expression of Nox-1 caused by MCT (Figure 5A).

### 3.7. Reverse Transcriptase Polymerase Chain Reaction (RT-PCR)

MCT administration significantly decreased mRNA expression of Bcl2/Bax ratio in RV as compared to control (2.5 fold; *p* < 0.001), suggestive of apoptotic changes which was preserved significantly by OS and sildenafil (*p* < 0.001) administration (Figure 5B).

## 4. Discussion

In the present study, we have evaluated the therapeutic potential of an aqueous extract of the leaves of OS in MCT-induced PH in rats and its effect on pulmonary oxidative stress markers and pulmonary vascular remodeling. A single dose of MCT led to increased right ventricular systolic pressure (RVSP) and right ventricular hypertrophy (RVH), as evidenced by the RV/LV + IVS (Fulton index) and RV/HW ratios. It also caused vascular smooth muscle hypertrophy of pulmonary arterioles in these rats. These findings established the development of PH, as also reported in earlier studies [44,45,46]. OS and sildenafil were started from day 29 to day 42. At day 28, echocardiography was done to assess the development of MCT-induced PH [44,45]. It was observed that OS and sildenafil significantly ameliorated the raised RVSP and RV hypertrophy caused by MCT. There is a variability of RVSP which has been reported in MCT-induced PH. Moreover, our data is also supported by some other groups [47,48].

It has been demonstrated that MCT-induced PH causes structural changes in pulmonary arteries which are similar to the characteristic features of human PH in terms of marked medial wall thickening resulting in a dramatic increase in pulmonary arterial resistance [37,49]. In our study, it has been observed that the sildenafil and OS decreased the thickness of smaller arterioles (<100 μm), an important pathological feature of PH. Moreover, it has been also reported that sildenafil prevented pulmonary arterial remodeling in rats and this effect might be due to vasodilatation and pulmonary vascular proliferation reduction [50]. Ours is the first study to show that OS has therapeutic potential against MCT-induced structural changes in pulmonary arteries. This effect of OS supports its disease modifying effect and has a significant clinical relevance. As it has been demonstrated that pulmonary vascular remodeling, a pathological feature of PH which leads to increased pulmonary vascular resistance [44,49], subsequent rise in pulmonary artery pressure, and increased RVSP as well as RV hypertrophy [50]. In addition, there was a significant increase in the ratio of lung weight to body weight in MCT administered rats which corroborates with earlier findings [51]. This was also significantly ameliorated with OS treatment, but sildenafil did not cause any significant difference.

It has been demonstrated that echocardiography is a non-invasive and a key screening tool in the diagnostic algorithm for PH in human and it is also used in rat models [52]. In the present study, MCT administration caused significant increase in right ventricular outflow tract/aortic outflow diameter (RVoTD/AoD) and decrease in pulmonary artery acceleration time/ejection time (PAAT/ET) ratio. It has been reported that these changes happen due to increased pulmonary artery resistance and subsequent RV hypertrophy [25,28]. In addition, we did not find any significant change in heart rate among the groups which is in agreement with the earlier study [27]. Ours is the first study to show the therapeutic effect of OS on both of these echocardiography parameters in MCT-induced PH. Sildenafil preserved only the ratio of RVoTD/AoD caused by MCT. It suggests that OS has therapeutic effect on both pulmonary artery remodeling and RV hypertrophy which leads to an increase in RVSP, while sildenafil had a curative effect on RV hypertrophy (RVH). OS could be beneficial to ameliorate RV failure caused by RVH due to pressure overload in PH.

It has been reported that oxidative stress plays an important role in the development and progression of PH. Increased oxidative stress has been also reported in the lungs of different rat models of PH [53]. Nicotinamide adenine dinucleotide phosphate (NADPH) oxidase (Nox) has been demonstrated to be the major source for reactive oxygen species (ROS) in MCT-induced PH. In addition, Nox-1 expression has been found to be increased in human and rat PH, respectively [54,55]. In the present study, MCT caused significant increase in the markers of oxidative stress like lung TBARS levels and decrease in reduced GSH levels, superoxide dismutase levels and catalase levels. OS treatment significantly decreased the lung TBARS levels and increased the lung catalase levels. This therapeutic effect of OS might be due to its antioxidant property. However, sildenafil only decreased the lung TBARS levels while OS and sildenafil did not cause significant difference in lung SOD levels and lung GSH levels. This might be due to the presence of a higher oxidative state which leads to consumption of antioxidants to neutralize free radicals. Moreover, OS treatment significantly preserved the lung Nox-1 protein levels as compared to control. Thus, the salutary effects of the OS are due to its antioxidant properties [1]. Moreover, it has been also reported that Nox-derived ROS are vital modulators of signal transduction pathways that control key physiological activities such as cell growth, proliferation, and apoptosis [56]. Therefore, the reduced level of Nox could be beneficial for RV hypertrophy. Chronic PH leads to RV hypertrophy which has been also observed in this study. RV hypertrophy led to right ventricular failure which is a major cause of morbidity and mortality in the patients of PH [15,57]. In addition, we observed that OS had an anti-apoptotic effect and preserved the decrease in Bcl2/Bax ratio in RV caused by MCT. The apparent anti-apoptotic effect manifested by OS might be useful in preventing RV failure which is a major cause of morbidity in PH.

It has been demonstrated that PH induced by a single pathological way, such as with MCT or chronic hypoxia, do not fully show the severity of PH observed in clinics with respect to histological and/or hemodynamic parameters [58]. It has been also suggested that a multiple pathological insult animal model could correlate better with severity of human PH [58]. Therefore, selection of only an MCT-induced animal model for PH might be a limitation of this study in the context of clinical PH.

In the present study, it has been demonstrated that OS improved major characteristic pathological features of MCT-induced PH in rats, including increased RVSP, right ventricular hypertrophy, echocardiographic parameters (increased RVoTD/AoD and reduced PAAT/ET), altered oxidative stress markers, increased expression of lung Nox-1 protein, proapoptotic changes in right ventricle, and most importantly, increased thickness of distal arterioles (of diameter <100 μm). It has been observed that OS has a certain therapeutic advantage over sildenafil, a commonly used drug for this condition.

## Figures and Tables

**Table 1:** Primers used for gene expression analysis.

Name	Direction	Sequences	Product Size	Temperature
BCL-2	Forward	CCATGACTGAGGGACCAACT	150 bp	56 °C
	Reverse	CTCTTCTTCCTGCCCTTCCT		
BAX	Forward	TGCAGAGGATGATTGCGACT	200 bp	60 °C
	Reverse	GATCAGCTCGGGCACTTTAG		
RPL32	Forward	AGATTCAAGGGCCAGATCCT	175 bp	57 °C
	Reverse	CGATGGCTTTTCGGTTCTTA		

**Table 2:** Effect of OS on lung thiobarbituric acid reactive substances (TBARS) levels, lung reduced GSH, lung catalase, and lung superoxide dismutase (SOD) levels in MCT-induced PH. Values are expressed as mean ± SEM (*n* = 6). * *p* < 0.05, ** *p* < 0.01, *** *p* < 0.001; a—as compared to control, b—as compared to MCT.

Groups	TBARS (nmol/mg protein)	GSH (mg/mg protein)	Catalase (U/mg protein)	SOD (U/mg protein)
Control	19.60 ± 2.5	27.36 ± 3.0	0.85 ± 0.04	110.51 ± 10.8
MCT	155.46 ± 13.4 ***^,a^	13.98 ± 2.0 ***^,a^	0.21 ± 0.01 ***^,a^	35.4 ± 5.7 ***^,a^
MCT + SIL	72.36 ± 12.7 **^,b^	17.22 ± 3.1	0.18 ± 0.02	51.98 ± 3.5
MCT + OS	99.05 ± 20.5 *^,b^	12.99 ± 1.9	0.51 ± 0.04 ***^,b^	41.49 ± 6.4

## References

[1] Singh V., Kahol A., Singh I.P., Saraf I., Shri R. (2016). Evaluation of anti-amnesic effect of extracts of selected *Ocimum* species using in-vitro and in-vivo models. J. Ethnopharmacol..

[2] Godhwani S., Godhwani J.L., Vyas D.S. (1988). *Ocimum sanctum*—A preliminary study evaluating its immunoregulatory profile in albino rats. J. Ethnopharmacol..

[3] Singh S., Majumdar D.K., Rehan H.M. (1996). Evaluation of anti-inflammatory potential of fixed oil of *Ocimum sanctum* (Holybasil) and its possible mechanism of action. J. Ethnopharmacol..

[4] Singh S., Taneja M., Majumdar D.K. (2007). Biological activities of *Ocimum sanctum* L. fixed oil—An overview. Indian J. Exp. Biol..

[5] Gupta S.K., Prakash J., Srivastava S. (2002). Validation of traditional claim of Tulsi, *Ocimum sanctum* Linn. as a medicinal plant. Indian J. Exp. Biol..

[6] Mohanty I., Arya D.S., Gupta S.K. (2006). Effect of Curcuma longa and *Ocimum sanctum* on myocardial apoptosis in experimentally induced myocardial ischemic-reperfusion injury. BMC Complement. Altern. Med..

[7] Singh S., Rehan H.M., Majumdar D.K. (2001). Effect of *Ocimum sanctum* fixed oil on blood pressure, blood clotting time and pentobarbitone-induced sleeping time. J. Ethnopharmacol..

[8] Sood S., Narang D., Dinda A.K., Maulik S.K. (2005). Chronic oral administration of *Ocimum sanctum* Linn. augments cardiac endogenous antioxidants and prevents isoproterenol-induced myocardial necrosis in rats. J. Pharm. Pharmacol..

[9] Uma Devi P., Ganasoundari A., Vrinda B., Srinivasan K.K., Unnikrishnan M.K. (2000). Radiation protection by the Ocimum flavonoids orientin and vicenin: Mechanisms of action. Radiat. Res..

[10] Mediratta P.K., Dewan V., Bhattacharya S.K., Gupta V.S., Maiti P.C., Sen P. (1988). Effect of *Ocimum sanctum* Linn. on humoral immune responses. Indian J. Med. Res..

[11] Suanarunsawat T., Ayutthaya W.D.N., Songsak T., Thirawarapan S., Poungshompoo S. (2011). Lipid-lowering and antioxidative activities of aqueous extracts of *Ocimum sanctum* L. leaves in rats fed with a high-cholesterol diet. Oxid. Med. Cell. Longev..

[12] Sood S., Narang D., Thomas M.K., Gupta Y.K., Maulik S.K. (2006). Effect of *Ocimum sanctum* Linn. on cardiac changes in rats subjected to chronic restraint stress. J. Ethnopharmacol..

[13] Manikandan P., VidjayaLetchoumy P., Prathiba D., Nagini S. (2007). Proliferation, angiogenesis and apoptosis-associated proteins are molecular targets for chemoprevention of MNNG-induced gastric carcinogenesis by ethanolic *Ocimum sanctum* leaf extract. Singap. Med. J..

[14] Simonneau G., Gatzoulis M.A., Adatia I., Celermajer D., Denton C., Ghofrani A., Gomez Sanchez M.A., Krishna Kumar R., Landzberg M., Machado R.F. (2013). Updated Clinical Classification of Pulmonary Hypertension. J. Am. Coll. Cardiol..

[15] Korsholm K., Andersen A., Kirkfeldt R.E., Hansen K.N., Mellemkjær S., Nielsen-Kudsk J.E. (2015). Survival in an incident cohort of patients with pulmonary arterial hypertension in Denmark. Pulm. Circ..

[16] Schermuly R.T., Ghofrani H.A., Wilkins M.R., Grimminger F. (2011). Mechanisms of disease: Pulmonary arterial hypertension. Nat. Rev. Cardiol..

[17] Rahman K. (2007). Studies on free radicals, antioxidants, and co-factors. Clin. Interv. Aging.

[18] Veit F., Pak O., Egemnazarov B., Roth M., Kosanovic D., Seimetz M., Sommer N., Ghofrani H.A., Seeger W., Grimminger F. (2013). Function of NADPH Oxidase 1 in Pulmonary Arterial Smooth Muscle Cells after Monocrotaline-Induced Pulmonary Vascular Remodeling. Antioxid. Redox Signal..

[19] Hood K.Y., Montezano A.C., Harvey A.P., Nilsen M., MacLean M.R., Touyz R.M. (2016). Nicotinamide Adenine Dinucleotide Phosphate Oxidase-Mediated Redox Signaling and Vascular Remodeling by 16α-Hydroxyestrone in Human Pulmonary Artery Cells: Implications in Pulmonary Arterial Hypertension. Hypertension.

[20] Li Q., Mao M., Qiu Y., Liu G., Sheng T., Yu X., Wang S., Zhu D. (2016). Key Role of ROS in the Process of 15-Lipoxygenase/15-Hydroxyeicosatetraenoiccid-Induced Pulmonary Vascular Remodeling in Hypoxia Pulmonary Hypertension. PLoS ONE.

[21] Paffett M.L., Lucas S.N., Campen M.J. (2012). Resveratrol Reverses Monocrotaline-Induced Pulmonary Vascular and Cardiac Dysfunction: A Potential Role for Atrogin-1 in Smooth Muscle. Vascul. Pharmacol..

[22] Zuckerbraun B.S., Chin B.Y., Wegiel B., Billiar T.R., Czsimadia E., Rao J., Shimoda L., Ifedigbo E., Kanno S., Otterbein L.E. (2006). Carbon monoxide reverses established pulmonary hypertension. J. Exp. Med..

[23] Okada M., Harada T., Kikuzuki R., Yamawaki H., Hara Y. (2009). Effects of telmisartan on right ventricular remodeling induced by monocrotaline in rats. J. Pharmacol. Sci..

[24] Kato Y., Iwase M., Kanazawa H., Kawata N., Yoshimori Y., Hashimoto K., Yokoi T., Noda A., Takagi K., Koike Y. (2003). Progressive Development of Pulmonary Hypertension Leading to Right Ventricular Hypertrophy Assessed by Echocardiography in Rats. Exp. Anim..

[25] Slama M., Susic D., Varagic J., Ahn J., Frohlich E.D. (2003). Echocardiographic measurement of cardiac output in rats. Am. J. Physiol. Heart Circ. Physiol..

[26] Thibault H.B., Kurtz B., Raher M.J., Shaik R.S., Waxman A., Derumeaux G., Halpern E.F., Bloch K.D., Scherrer-Crosbie M. (2010). Noninvasive assessment of murine pulmonary arterial pressure: Validation and application to models of pulmonary hypertension. Circ. Cardiovasc. Imaging.

[27] Kosanovic D., Kojonazarov B., Luitel H., Dahal B.K., Sydykov A., Cornitescu T., Janssen W., Brandes R.P., Davie N., Ghofrani H.A. (2011). Therapeutic efficacy of TBC3711 in monocrotaline-induced pulmonary hypertension. Respir. Res..

[28] Jones J.E., Mendes L., Rudd M.A., Russo G., Loscalzo J., Zhang Y.-Y. (2002). Serial noninvasive assessment of progressive pulmonary hypertension in a rat model. Am. J. Physiol. Heart Circ. Physiol..

[29] Werchan P.M., Summer W.R., Gerdes A.M., McDonough K.H. (1989). Right ventricular performance after monocrotaline-induced pulmonary hypertension. Am. J. Physiol..

[30] Girgis R.E., Li D., Zhan X., Garcia J.G.N., Tuder R.M., Hassoun P.M., Johns R.A. (2003). Attenuation of chronic hypoxic pulmonary hypertension by simvastatin. Am. J. Physiol. Heart Circ. Physiol..

[31] Silverton N., Meineri M., Djaiani G. (2015). The controversy of right ventricular systolic pressure: Is it time to abandon the pulmonary artery catheter?. Anaesthesia.

[32] Brunner F., Wölkart G., Haleen S. (2002). Defective intracellular calcium handling in monocrotaline-induced right ventricular hypertrophy: Protective effect of long-term endothelin-A receptor blockade with 2-benzo[1,3]dioxol-5-yl-3-benzyl-4-(4-methoxy-phenyl-)-4-oxobut-2-enoate-sodium (PD 155080). J. Pharmacol. Exp. Ther..

[33] Falcao-Pires I., Gonçalves N., Henriques-Coelho T., Moreira-Gonçalves D., Roncon-Albuquerque R., Leite-Moreira A.F. (2009). Apelin decreases myocardial injury and improves right ventricular function in monocrotaline-induced pulmonary hypertension. Am. J. Physiol. Heart Circ. Physiol..

[34] Ma Z., Mao L., Rajagopal S. (2016). Hemodynamic Characterization of Rodent Models of Pulmonary Arterial Hypertension. J. Vis. Exp..

[35] Cilley R.E., Wang J.Y., Coran A.G. (1993). Lung injury produced by moderate lung overinflation in rats. J. Pediatr. Surg..

[36] Ito T., Okada T., Miyashita H., Nomoto T., Nonaka-Sarukawa M., Uchibori R., Maeda Y., Urabe M., Mizukami H., Kume A. (2007). Interleukin-10 expression mediated by an adeno-associated virus vector prevents monocrotaline-induced pulmonary arterial hypertension in rats. Circ. Res..

[37] Prié S., Leung T.K., Cernacek P., Ryan J.W., Dupuis J. (1997). The orally active ET(A) receptor antagonist (+)-(S)-2-(4,6-dimethoxy-pyrimidin-2-yloxy)-3-methoxy-3,3-diphe nyl-propionic acid (LU 135252) prevents the development of pulmonary hypertension and endothelial metabolic dysfunction in monocrotaline-treated rats. J. Pharmacol. Exp. Ther..

[38] Kay J.M., Keane P.M., Suyama K.L., Gauthier D. (1982). Angiotensin converting enzyme activity and evolution of pulmonary vascular disease in rats with monocrotaline pulmonary hypertension. Thorax.

[39] Ohkawa H., Ohishi N., Yagi K. (1979). Assay for lipid peroxides in animal tissues by thiobarbituric acid reaction. Anal. Biochem..

[40] Ellman G.L. (1959). Tissue sulfhydryl groups. Arch. Biochem. Biophys..

[41] Aebi H. (1984). Catalase in vitro. Methods Enzymol..

[42] Kakkar P., Das B., Viswanathan P.N. (1984). A modified spectrophotometric assay of superoxide dismutase. Indian J. Biochem. Biophys..

[43] Banerjee S.K., Wang D.W., Alzamora R., Huang X.N., Pastor-Soler N.M., Hallows K.R., McGaffin K.R., Ahmad F. (2010). SGLT1, a novel cardiac glucose transporter, mediates increased glucose uptake in PRKAG2 cardiomyopathy. J. Mol. Cell. Cardiol..

[44] Meghwani H., Prabhakar P., Mohammed S.A., Seth S., Hote M.P., Banerjee S.K., Arava S., Ray R., Maulik S.K. (2017). Beneficial effects of aqueous extract of stem bark of *Terminalia arjuna* (Roxb.), An ayurvedic drug in experimental pulmonary hypertension. J. Ethnopharmacol..

[45] Boissiere J., Gautier M., Machet M.-C., Hanton G., Bonnet P., Eder V. (2005). Doppler tissue imaging in assessment of pulmonary hypertension-induced right ventricle dysfunction. Am. J. Physiol. Heart Circ. Physiol..

[46] Qin N., Gong Q.H., Wei L.W., Wu Q., Huang X.-N. (2008). Total ginsenosides inhibit the right ventricular hypertrophy induced by monocrotaline in rats. Biol. Pharm. Bull..

[47] Bal E., Ilgin S., Atli O., Ergun B., Sirmagul B. (2013). The effects of gender difference on monocrotaline-induced pulmonary hypertension in rats. Hum. Exp. Toxicol..

[48] Kato T., Nasu T., Sonoda H., Ito K.M., Ikeda M., Ito K. (2008). Evaluation of olmesartan medoxomil in the rat monocrotaline model of pulmonary hypertension. J. Cardiovasc. Pharmacol..

[49] Jeffery T.K., Wanstall J.C. (2001). Pulmonary vascular remodeling: A target for therapeutic intervention in pulmonary hypertension. Pharmacol. Ther..

[50] Bogdan S., Seferian A., Totoescu A., Dumitrache-Rujinski S., Ceausu M., Coman C., Ardelean C.M., Dorobantu M., Bogdan M. (2012). Sildenafil reduces inflammation and prevents pulmonary arterial remodeling of the monocrotaline-induced disease in the wistar rats. Maedica.

[51] Burch G.H., Jensen L.R., Pappas J., Hammond E.H., Banner W., Shaddy R.E. (1996). Growth factor expression and effects of amrinone in monocrotaline-induced pulmonary hypertension in rats. Biochem. Mol. Med..

[52] Koskenvuo J.W., Mirsky R., Zhang Y., Angeli F.S., Jahn S., Alastalo T.P., Schiller N.B., Boyle A.J., Chatterjee K., De Marco T. (2010). A comparison of echocardiography to invasive measurement in the evaluation of pulmonary arterial hypertension in a rat model. Int. J. Cardiovasc. Imaging.

[53] Demarco V.G., Whaley-Connell A.T., Sowers J.R., Habibi J., Dellsperger K.C. (2010). Contribution of oxidative stress to pulmonary arterial hypertension. World J. Cardiol..

[54] Ghouleh I.A., Sahoo S., Meijles D.N., Amaral J.H., de Jesus D.S., Sembrat J., Rojas M., Goncharov D.A., Goncharova E.A., Pagano P.J. (2017). Endothelial Nox1 oxidase assembly inhuman pulmonary arterial hypertension; driver of Gremlin1-mediated proliferation. Clin. Sci..

[55] Csiszar A., Labinskyy N., Olson S., Pinto J.T., Gupte S., Wu J.M., Hu F., Ballabh P., Podlutsky A., Losonczy G. (2009). Resveratrol prevents monocrotaline-induced pulmonary hypertension in rats. Hypertension.

[56] Manea S.A., Constantin A., Manda G., Sasson S., Manea A. (2015). Regulation of Nox enzymes expression in vascular pathophysiology: Focusing on transcription factors and epigenetic mechanisms. Redox Biol..

[57] McLaughlin V.V., Davis M., Cornwell W. (2011). Pulmonary arterial hypertension. Curr. Probl. Cardiol..

[58] Maarman G., Lecour S., Butrous G., Thienemann F., Sliwa K. (2013). A comprehensive review: The evolution of animal models in pulmonary hypertension research; are we there yet?. Pulm. Circ..

